# Quantum probability assignment limited by relativistic causality

**DOI:** 10.1038/srep22986

**Published:** 2016-03-14

**Authors:** Yeong Deok Han, Taeseung Choi

**Affiliations:** 1 Department of Computer Science and Engineering, Woosuk University, Wanju, Cheonbuk, 565-701, Korea; 2 Division of Applied Food System, College of Natural Science, Seoul Women’s University, Seoul 139-774, Korea; 3 School of Computational Sciences, Korea Institute for Advanced Study, Seoul 130-012, Korea

## Abstract

Quantum theory has nonlocal correlations, which bothered Einstein, but found to satisfy relativistic causality. Correlation for a shared quantum state manifests itself, in the standard quantum framework, by joint probability distributions that can be obtained by applying state reduction and probability assignment that is called Born rule. Quantum correlations, which show nonlocality when the shared state has an entanglement, can be changed if we apply different probability assignment rule. As a result, the amount of nonlocality in quantum correlation will be changed. The issue is whether the change of the rule of quantum probability assignment breaks relativistic causality. We have shown that Born rule on quantum measurement is derived by requiring relativistic causality condition. This shows how the relativistic causality limits the upper bound of quantum nonlocality through quantum probability assignment.

Quantum mechanics has nonlocal correlations to cause Einstein discomfort by a spooky action at a distance^1^,^2^. Even though quantum correlations show nonlocality, they do not violate relativistic causality. Quantum nonlocal correlations are demonstrated in measurements on an entangled state shared between two space-like separate parties, Alice and Bob^3^. A local measurement on one of the entangled pair by Alice (Bob) reduces the other state of Bob (Alice) instantaneously in the standard quantum physics. Quantum theory is not deterministic theory, which has probabilistic outcomes in the measurement through the reduction of a quantum state into an eigenstate of an observable. The state reduction and the probability assignment for the measurement outcomes will determine quantum correlations.

The instantaneous reduction of the entangled state can be explained by a hypothetical influence with infinite speed between two space-like separate parties. Recent experiments determined that the lower bound of the speed of the hypothetical influence has to exceed the speed of light by at least four orders of magnitude, and suggest that the speed of the hypothetical influence would be infinite^4^,^5^. However, the experiment cannot determine whether the speed of the hypothetical influence is infinite, but can only specify lower bound of the hypothetical influence. Bancal *et al*. have shown theoretically that for any finite speed hypothetical influences, faster-than-light communication can be built^6^. According to their results, only when the speed of the hypothetical influence is infinite, the quantum nonlocality cannot be used as a tool for faster-than-light signaling, which violates relativistic causality.

The measurement postulate in the standard quantum mechanics states that the probability assignment to measurement outcomes is governed by Born rule^7^. Quantum correlations, obtained by applying Born rule to a shared entangled quantum state, show nonlocality^8^. The amount of nonlocality can be demonstrated by a violation of the Clauser-Horne-Shimony-Holt (CHSH) inequality, bounded by 2 in any local classical theory^9^. The upper bound of quantum correlations, which is known as Tsirelson’s bound, is 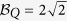
10. Popescu and Rohrlich found that nonlocal binary devices with a certain joint probability distributions can reach the maximum upper bound 4 under no faster-than-light signaling condition, required by relativistic causality^11^. As a result, they have shown the existence of ‘superquantum’ correlations that are more nonlocal than quantum correlations under relativistic causality. Several attempts to explain the reason why post-quantum theory, which has superquantum correlations, was not found in nature have been proposed^12^,^13^,^14^,^15^,^16^,^17^. However, this is still an open question. The nonlocality of quantum mechanics can be increased by assigning other quantum probabilities on measurement outcomes but this assignment may break relativistic causality. Here we ask a question differently, “Can Born rule in quantum mechanics be derived by relativistic causality?”.

In general the causality requirement has been considered as a prohibition of faster-than-light signaling, which is called ‘no-signaling’ condition. However, no-signaling condition is not enough to determine the specific form of probability assignment on local measurements (Methods). Hence another form of relativistic causality will be considered here. That causality condition is related with nonexistence of time ordering between space-like separate events. In special relativity, the time sequence of any two space-like separated events for one inertial observer could be changed according to the motion of different inertial observers. This means that there is no absolute time order between any two space-like separated events, which all observers agree on. Hence cause and its effect relation between space-like separated events are not possible because a causal relation requires absolute time ordering. This causality, which requires no causal relation between two space-like separate events, is usual causality, however, to distinguish this causality from no-signaling condition, we will call it ‘space-like causality’ condition. The space-like causality condition is satisfied in the standard quantum framework with the fact that joint probabilities of space-like separate measurements on a composite state are independent on time ordering of the measurements^8^. We will show that Born rule is the unique probability assignment rule on quantum measurement by using the space-like causality condition.

## Results

### Derivation of Born rule

To derive Born rule, we first generalize quantum probability assignment from Born rule, while maintaining other quantum postulates in the standard textbook unchanged, and then investigate its consequence under space-like causality condition. In the standard quantum framework^18^, a physical observable 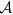 is a linear Hermitian operator with real eigenvalues 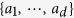 and mutually orthonormal eigenvectors 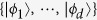, where *d* is the dimension of a separable Hilbert space. Then a general quantum state 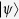 is represented as a linear superposition of eigenstates. Physical observables satisfy the following measurement postulates: i) an outcome of a measurement is always an eigenvalue of 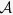. ii) The probability of an outcome *a*_*k*_ for the initial state 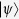 is obtained with 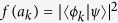. iii) The quantum state after the measurement that gives the outcome *a*_*k*_ reduces to the corresponding eigenstate 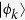. The modification of postulate i) has nothing to do with relativistic causality because nonlocal correlations are implemented by an outcome probability not by the value of an outcome. The modification of postulate iii) is not desirable because it is natural for physical systems that sequential measurements without any perturbation would give the same measurement results for the same observable 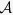.

The postulate iii) needs further explanation when *a*_*k*_ is a degenerate eigenvalue of the observable 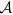
8. In the degenerate case, the eigenstates of the observable 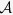 form a subspace whose dimension is called degeneracy. This means that the outcome of the observable 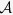 cannot uniquely determine the corresponding eigenstate of the observable 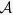, because the eigenstate of the observable 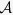 can be any normalized state in the subspace. In the degenerate case, we can always choose another observable 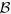, which commutes with the observable 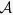, to resolve the degeneracy of the observable 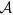. Here we assume that the observable 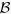 resolves all the degeneracies for simplicity without lack of generality. Then a general initial state 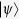 is written as 
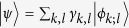
, where *l* goes from 1 to the degeneracy *d*_*k*_, which depends on *k* in general. The state 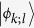 are simultaneous eigenstates of 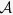 and 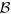 such that 
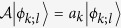
 and 
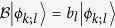
. Then the initial state 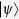 can be rewritten as


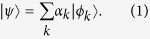


Here the normalized states 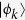 and the coefficients *a*_*k*_ are


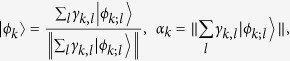


where 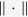 denotes the norm of a state in a Hilbert space. After the measurement of the observable 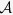 with outcome *a*_*k*_ on 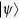, the state must reduce to 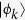. One can check that the commutativity between two observables 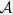 and 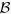 is not satisfied if the reduced state after measurement becomes another linear combination state 
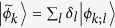
 different from the state 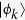 in the initial state 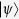. These arguments are also valid for another observable 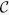 to resolve all the degeneracies of the observable 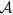. The simple example is that 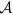 is *S*^2^, 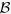 is *S*_*z*_, and 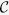 is *S*_*x*_ for spin problems, where *S*^2^ is total spin angular momentum operator squared, and *S*_*z*_ and *S*_*x*_ are *z*- and *x*-component of spin angular momentum operator, respectively. Notice that the reduced state of the initial state 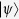 after the measurement with 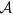 does not depend on whether the basis of subspace are eigenstates of 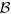 or 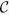. Now we will focus on a generalization of the quantum probability assignment of postulate ii), which is known as Born rule, under the constraint of relativistic causality.

In the standard quantum framework, all measurements are assumed to be local, however, the joint probability distributions of local measurements for a composite state shared by space-like separated parties could show nonlocal correlations. Hence a generalization of Born rule, which gives probability of a local measurement outcome, will change nonlocality such that joint probability distributions given by generalized probability assignment could break relativistic causality. We will consider a bipartite state shared by space-like separate parties, Alice and Bob, as a nonlocal device. If the shared state is a separable state, it trivially satisfy space-like causality because separable state gives no correlation between two parties. Hence it is required to consider an entangled state, and it is enough to consider a pair of entangled qubits because this is a minimal case to have a nonlocal correlation between two parties.

Since the joint probability distributions for an entangled qubits are determined by local measurement probabilities on each shared state, it is enough to define generalized probability assignment rule on a state in a two-dimensional Hilbert space for investigating the consequence of new nonlocal correlations generated by generalized probability assignment rule. Let us define a generalized probability assignment rule on a state in a two-dimensional Hilbert space. We consider the qubit, which is given by the state 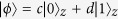, where 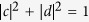 and 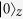 and 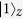 denote eigenvectors corresponding to eigenvalues 0 and 1 of an input (observable) *z*, respectively. For our purpose, it is enough to consider a pure state, because the mixed state is just a statistical mixture of pure states. The probability assignment for quantum measurement on the state 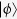 can be generalized from Born rule by applying an arbitrary non-negative real function *H*(*c*) of complex number *c* to the measurement probability 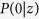 of the outcome 0 for the input *z*, i.e., 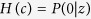. The other measurement probability 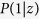 for the same input *z* and the outcome 1 can be determined by the normalization of probability as 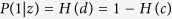. Note that *H*(0) = 0 and *H*(*u*) = 1, where *u* is a unit modulus complex number, because the initial states in these cases are described by one eigenvector. Born rule corresponds to 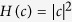.

We will show that Born rule is derived by imposing space-like causality condition to new quantum correlations generated by the generalized quantum probability assignment *H*(*c*) on local measurement outcomes of entangled qubits. Here we assume the non-negative function *H*(*c*) as a function 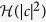 of the absolute value squared 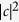 for clear understanding of the essential context. The derivation for the general non-negative real function *H*(*c*) of *c* is given in Methods. Space-like causality condition requires that joint probability distributions for space-like separate measurements should not depend on time ordering of local measurements. The joint probability distributions given by Born rule are independent on time ordering of local measurements, hence the standard quantum measurement postulates satisfy the space-like causality condition^7^. However joint probability distributions, which depend on time ordering of local measurements on a nonlocal device, can be constructed in general.

In special relativity, time ordering between space-like separated measurement events is not absolute. That is, the time sequence of two space-like events for one inertial observer could be inverted for another inertial observer moving with respect to the first observer. This implies that there is no absolute global time, on which every observer agrees. However, even though there is no absolute global time, observer-dependent global time can be well-defined. Hence we will consider the time ordering of joint probability distributions for an inertial observer *O*, who observes space-like separated measurements of Alice and Bob on a pair of bipartite entangled qubits with input (observables) *x* and *y*, respectively. The correlations between two qubits are described by joint probability distributions 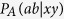 or 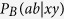 depending on the time ordering of Alice’s and Bob’s measurements in *O*’s reference frame. 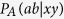 represents that Alice’s measurement precedes Bob’s measurement (Alice-first measurement) and similar to 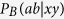. Here *a* (*b*) is the outcome of Alice’s (Bob’s) measurement with the input *x* (*y*). For the observer *O*, the temporal order of measurements of Alice and Bob is clearly determined in *O*’s own reference frame. The choice of one observer cause no problem against space-like causality because we finally require that the joint probability distributions should not depend on the time ordering of any observer including the observer *O*. The space-like causality condition requires that the joint probability distributions of space-like separated measurement events have to satisfy


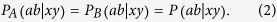


In quantum mechanics, a minimal nonlocal bipartite device is a pair of entangled qubits. Let us suppose that Alice and Bob are at rest in *O*’s reference frame. Alice and Bob share the following general state for a pair of entangled qubits described by


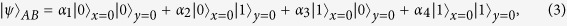


where 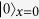, 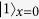, 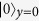, and 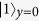 are the eigenstates of Alice’s input *x* = 0 and Bob’s input *y* = 0, respectively. In the derivation of Born rule, we will only use one kind of input so the notation with subscripts seems to be not necessary, but it will be used in Methods to investigate no-signaling condition. We will denote 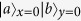 simply as 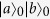. In fact, the state 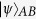 describes all the states of a pair of qubits including separable state with arbitrary complex numbers satisfying 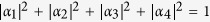.

It is enough to investigate the joint probability of outcomes (0, 0) for inputs (0, 0) because of relabeling symmetry of input and outcome of a qubit. In usual case, quantum nonlocal correlations are studied by using joint probability distributions with different measurement settings (input observables) as the study for no-signaling condition in Methods. In our derivation, we instead consider the order of measurements by each parties. The results of changing the order of measurement will show similar effect to different measurement settings by one party. As a simple example, let us consider the Bell state


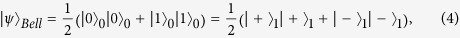


where the states 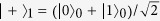 and 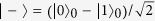 are eigenstates of input 1, respectively. Let us suppose that the input of Alice’s measurement is 0 and Bob’s 1. Then the joint probability distributions of Bob-first measurement on 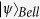 can be reproduced by those of Alice-first measurement with different measurement settings of Alice’s input 1 and Bob’s input 0.

Now let us first calculate the joint probability *P*_*A*_(00|00) of Alice-first measurement. The initial state 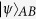 is a 4-dimensional vector not a two-dimensional vector so that there seems to have a problem to apply the generalized quantum probability assignment 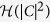, defined for a qubit, to Alice’s measurement. The locality in special relativity is commonly accepted by the commutativity of space-like separated observables^19^. Hence the observables of Alice’s input *x* = 0 and Bob’s input *y* = 0 commute each other and the Alice’s outcome *a* = 0 can be considered as degenerate in Alice-first measurement. As in Eq. (1), we can rewrite the state 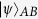 by taking out the common factor of the eigenvectors of Alice’s input *x* = 0 as


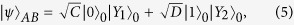


where 
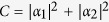
 and 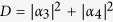. 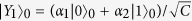 and 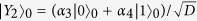 are normalized states of Bob’s qubit. Then the two vectors 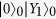 and 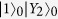 are orthonormal and form a two-dimensional Hilbert space so that the state 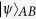 is a vector in this two-dimensional Hilbert space. Hence Alice’s measurement as the first measurement can be considered as a measurement on a vector in two-dimensional Hilbert space. After Alice’s first measurement, the state 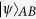 collapses either to 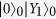 or to 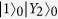 corresponding to an outcome 0 or 1, with the probabilities determined by the generalized probability assignment. That is, the state of Bob, after the measurement of Alice with input *x* = 0 and outcome 0, is projected to 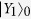 with probability 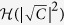. Then the probability of outcome 0 for Bob’s later measurement on the state 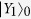 with input *y* = 0 is determined by 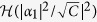, hence the joint probability of a pair of outcomes (0, 0) for a pair of inputs (0, 0) of Alice and Bob in the Alice-first measurement is obtained by the product of 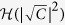 and 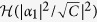, i.e.,


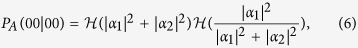


where we used 
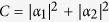
.

Now let us consider Bob-first measurement, in which the following factorization of the state 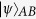 is necessary,


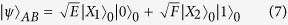


with 
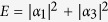
, 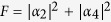, 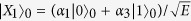, and 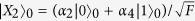. By a similar calculation to Alice-first measurement, the joint probability of Bob-first measurement for the same inputs (0, 0) and outputs (0, 0) as the Alice-first measurement can be obtained as


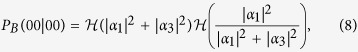


applying the same generalized probability assignment to Bob-first measurement.

Space-like causality condition, which requires 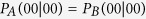, gives the relation


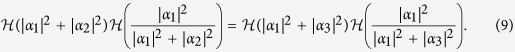


This relation should be satisfied for arbitrary *α*_1_, *α*_2_, *α*_3_, and *α*_4_. By substituting 0 for *α*_3_, the equality of Eqs (6) and (8) gives the following relation


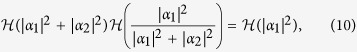


because 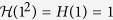. The above relation also has to be satisfied when *α*_1_ and *α*_2_ are exchanged with each other because of the freedom of relabeling outcomes 0 ↔ 1. And then we obtain the relation


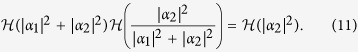


By adding those two relations in Eqs (10) and (11) we obtain


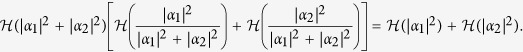


The addition of two probabilities, 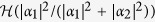 and 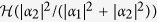, becomes 1 from the probability normalization because the sum of two arguments 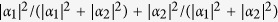 is 1. Finally we get the following relation


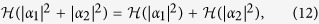


which requires that the functional form of 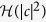 should be linear. Considering the probability normalization, 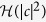 is determined as 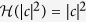, which is exactly Born rule. It can be shown that a general probability assignment *H*(*c*) is also limited to Born rule 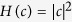 under the space-like causality condition as in Methods. In consequence, we have derived Born rule as the unique quantum probability assignment of measurements on qubits, which is consistent with relativistic causality. The derivation of Born rule in a higher dimensional Hilbert space will be essentially the same as the derivation in two-dimensional Hilbert space because the Hilbert space of the minimal case can always be considered as the subspace of higher dimensional Hilbert space.

As a reference, we briefly show, in Methods, that no-signaling condition cannot determine a specific form for the general quantum probability assignment 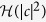.

## Discussion

In this paper, we have shown that Born rule in the standard quantum theory is the only possibility for assigning the probabilities to measurement outcomes on quantum states, which satisfies the relativistic causality. Several authors have derived Born rule in another approaches^20^,^21^. Gleason used non-contextuality to prove Born rule in the Hilbert space with dimension greater than two, and Zurek suggested the new symmetry ‘envariance’ which is the entanglement induced invariance to derive the Born rule^22^. Their derivations have some implications to understand quantum theory, and our derivation of Born rule implies that there is a profound relationship between quantum theory and relativity through measurement.

The pair of qubits shared by two space-like separate parties is minimal models to show nonlocal correlations, hence this model is enough to investigate the limit on the probability assignment of quantum measurement by relativity. Note that one can always choose two orthogonal vectors to use as a qubit, at least mathematically, in higher dimensional Hilbert space. No-signaling condition is shown not tight enough to derive Born rule, but space-like causality condition can successfully derive Born rule. Our derivation provides the understanding how relativity limits the nonlocal correlations of the quantum theory described in Hilbert space through measurement probability assignment. The fact that only Born rule is consistent with relativistic causality suggests that it is improbable to obtain a post-quantum theory by simply modifying the standard quantum theory. By this work, we hope to give a hint to understand the question of “Why is not quantum theory more nonlocal?”.

## Methods

### Derivation of Born rule for general probability assignment function *H*(*c*)

We will prove that 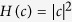 by considering the space-like causality condition of 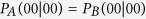. To consider *H*(*c*) as a function of *c* not of 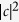, the initial state 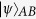 suitable for Alice-first measurement should be rewritten as


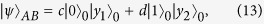


where 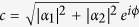, and 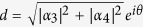. The states 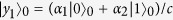 and 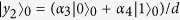 are easily checked to have unit norms. Then


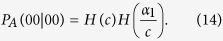


The useful description of the initial state for Bob-first measurement is


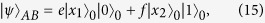


where 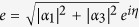, and 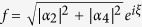. The states 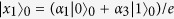 and 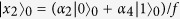 are normalized states. Then


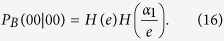


If we let *α*_3_ = 0, the space-like causality condition 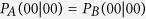 becomes


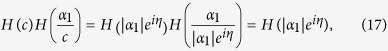


where we have used 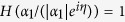 because *H*(*u*) = 1 for a uni-modular complex number *u*. Using *α*_1_ and *α*_2_ exchange symmetry, the relation in Eq. (17) becomes


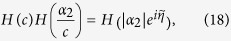


where the argument 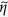 is defined similar to *η*.

By addition of two Eqs (17) and (18), we obtain


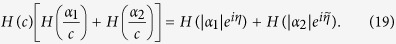


Because 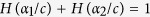, the above relation becomes


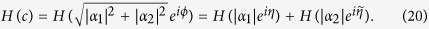


This equation must satisfy for arbitrary *α*_1_, *α*_2_, *ϕ*, *η*, and 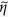 so that by letting *α*_1_ = 0 we obtain


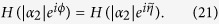


To satisfy this relation for arbitrary *ϕ* and 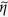, the function *H*(*c*) of *c* should not depend on the argument of the complex number *c*, but the absolute value 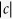. Eq. (20) requires that the functional form of *H*(*c*) should be 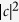, which is Born rule. Q.E.D

### No-signaling condition for general probability assignment

We suppose the situation that Alice is trying to send an information about her measurement inputs to Bob by choosing her inputs between 0 and 1 in her measurement, and Bob is trying to receive her information by measuring his outcome 0 for his input 0. The no-signaling condition in our denotation requires that


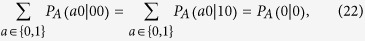


where 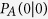 is the marginal conditional probability that Bob gets his outcome 0 for his input 0. This marginal probability is independent on the choice of Alice’s inputs in Eq. (22). To study no-signaling condition, we have to consider Alice’s another input *x* = 1. By using eigenvectors 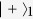 and 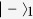 of the observable *x* = 1, the state 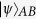 is rewritten as


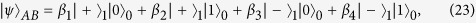


where 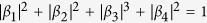. The relations among coefficients *α*_*i*_ and *β*_*j*_, where *i* and *j* are from 1 to 4, are obtained


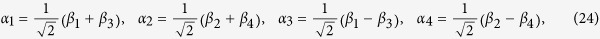


by using 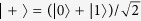 and 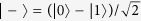. Then the no-signaling condition in Eq. (22) gives the relation


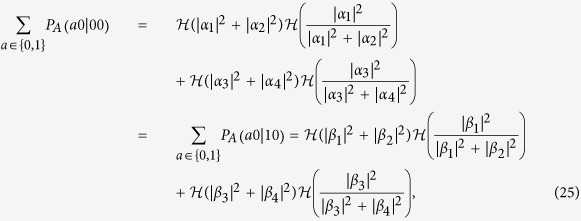


where 
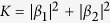
 and 
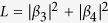
. This relation cannot determine the explicit form of 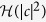. Notice that the relation in Eq. (9) from space-like causality is between one term of probability, but the relation in Eq. (25) from no-signaling condition is between summation of terms of probabilities. One can easily check, however, the relation in Eq. (25) holds for Born rule, i.e., 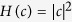, because 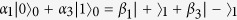.

## Additional Information

**How to cite this article**: Han, Y. D. and Choi, T. Quantum probability assignment limited by relativistic causality. *Sci. Rep*. **6**, 22986; doi: 10.1038/srep22986 (2016).
